# Relationship between leptin and white blood cells: a potential role in infection susceptibility and severity—the Olivetti Heart Study

**DOI:** 10.1007/s11739-023-03313-9

**Published:** 2023-05-22

**Authors:** Lanfranco D’Elia, Maria Masulli, Roberto Iacone, Ornella Russo, Pasquale Strazzullo, Ferruccio Galletti

**Affiliations:** Department of Clinical Medicine and Surgery, “Federico II” University of Naples Medical School, Via S. Pansini, 5, 80131 Naples, Italy

**Keywords:** Leptin, Adipocytokines, White blood cells, Immunity system, Infection severity, SARS-CoV-2

## Abstract

A number of evidence showed an emerging role of leptin on immune system, involving inflammation, and innate and adaptive immunity. Few observational studies have evaluated the relationship between leptin and immunity, albeit with low statistical power and methodological differences. Therefore, the aim of this study was to evaluate the potential role of leptin on the immunity, expressed as white blood cells (WBC)—and its subpopulations, by comprehensive multivariate models in a sample of adult men. A cross-sectional evaluation of a general population comprised 939 subjects participating in the Olivetti Heart Study, with available leptin levels and WBC—and its subpopulations. WBC were significantly and positively associated with leptin, C-reactive protein and HOMA index (*p* < 0.05), but not with age and anthropometric indices (*p* > 0.05). The multivariate analysis confirmed the association between leptin and WBC, after accounting for main confounders (*p* < 0.05). Additional analysis on WBC subpopulations showed a positive and significant correlation between leptin and lymphocytes, monocytes and eosinophils (*p* < 0.05), but not with neutrophils and basophils (*p* > 0.05). After stratification by body weight, the positive and significant association between leptin and WBC—and its subpopulations—was found in excess body weight participants. The results of this study indicate a direct relationship between leptin levels and WBC—and its subpopulations—in excess body weight participants. These results support the hypothesis that leptin has modulatory functions on immunity and role in the pathophysiology of immune-related diseases, in particular in those associated with excess body weight.

**Supplementary Information:**

The online version contains supplementary material available at 10.1007/s11739-023-03313-9.

## Introduction

Leptin is a peptidic hormone mainly synthesized by adipocytes in white adipose tissue in response to food intake and energy balance [1]. Indeed, it plays a crucial role in the regulation of body weight by suppression of the desire for food and the increment of energy expenditure [1].

However, circulating leptin levels are proportional to the body fat, hence individuals with excess body weight have high leptin levels [1, 2], suggesting ineffective metabolic actions of leptin (i.e., leptin resistance) [3]. Nevertheless, our previous studies revealed a positive association between leptin levels and cardiovascular and metabolic risk independent of body weight [4–8].

In addition, a number of evidence showed an emerging role of leptin on immune system, through the mediation of inflammation, hematopoiesis, and regulation of innate and adaptive immunity [9, 10].

Indeed, leptin increases during chronic inflammation [11] and has been recognized as one of the mediator of the inflammatory condition in excess body weight individuals [12, 13]. Moreover, circulating leptin levels also increase in acute inflammation and infection [9, 14]. In particular, in response to acute pneumonia and intestinal infection, there is an increased concentration of leptin in lung and intestinal epithelium, in addition to those of adipocytes and immune cells [15, 16]. Although this evidence, few observational studies in different settings have been performed to assess the relationship between leptin and immune system [17–21]. Nevertheless, the results of these studies are not consistent, because of the low statistical power of most studies, the heterogeneous studies characteristics and methodological differences. Recently, this issue has been object of interest, because of the amount of data about the role of the leptin on the immunity modulation [9, 11], and its especially involvement in excess body weight individuals during SARS-CoV-2 infection [9, 22–24].

We thus sought to analyze the association between circulating leptin levels and white blood cells (WBC), and its subpopulations, in a large sample of men participating in the Olivetti Heart Study (OHS).

## Methods

### Study population

The OHS was an occupational investigation of the male workforce of the Olivetti factories in Southern Italy (Pozzuoli-Naples and Marcianise-Caserta), as previously described [25, 26] (Online Resource 1). A total of 994 adult individuals was examined at 2002–04 visit. For the purposes of the present analysis, we excluded participants whose demographic and anthropometric characteristics, or leptin levels or WBC were not available (*n* = 55). Finally, this evaluation was performed on 939 participants. The Ethics Committee of “Federico II” University in Naples approved the Olivetti study protocol and the participants provided their informed written consent to participate.

### Examination procedures

The OHS study procedures have been described previously [25, 26]. Briefly, a physical examination was performed with the participants having fasted for at least 13 h. The visit included the administration of a questionnaire, a physical examination, anthropometric measurements and a blood test.

A fasting venous blood sample was taken in the seated position. Blood specimens were immediately centrifuged and stored at − 70 °C until analysis. WBC and its subpopulations were measured by an automated blood cell counter. Serum leptin levels were measured by an enzyme-linked immunosorbent assay (R&D System GmbH, Wiesbaden-Nordenstadt, Germany). Intra- and inter-assay coefficients of variation were 3.0 and 5.4%, respectively [27]. High-sensitivity C-reactive protein (CRP) was assessed by an immunoturbidimetric method (Roche Diagnostics, Milan, Italy, automated analyzer). Serum glucose levels were measured with automated methods (Cobas-Mira, Roche, Italy). Serum insulin was determined by radioimmunoassay (Insulin Lisophase; Technogenetics, Milan, Italy). Insulin sensitivity was estimated by the homeostasis model assessment (HOMA index) using the formula: fasting plasma insulin (μU/mL) x fasting plasma glucose (mmol/L)/22.5. A HOMA index > 2.77 UI was considered as a cutoff value for insulin resistance (IR).

Serum creatinine was measured by the picric acid colorimetric method. Estimated glomerular filtration rate (eGFR) was estimated by standard formula [28]. Renal damage was defined as eGFR lower than 60 ml/min/1.73 m^2^.

The questionnaire classified participants into current smokers, never smokers and ex-smokers. Body weight, height and waist circumference (WC) were measured as previously described (25, 26). Body mass index (BMI) was measured according to the formula weight (kg)/height^2^ (m). Excess body weight was defined as a BMI ≥ 25 kg/m^2^. Abdominal obesity was defined as a WC ≥ 102 cm.

Body adiposity index (BAI) was measured using the formula [29]:

[hip circumference (cm)/height1.5 (m)] − 18.

### Statistical analysis

All statistical analyses were performed using the SPSS software, version 23 (SPSS inc, Chicago, Ill).

As the distribution of WBC (and its subpopulations), leptin, HOMA index and CRP were skewed, log-transformed values were used for the analyses. Bivariate relationships between the variables under investigation were evaluated by Pearson’s correlation analysis. Moreover, the participants were also stratified according to body weight (i.e., normal-weight and excess body weight). Unpaired *t* test was used to assess the differences between normal-weight and excess body weight participants. The Chi-squared test was used to evaluate differences between categorical variables. A multivariable linear regression analysis was carried out to determine the independent effect of leptin per 1-standard deviation (SD, 2.1 ng/ml) on WBC and its subpopulations, adjusting for the main potential confounders. Given the strong statistical and physiological relationship between anthropometric indices (BMI and WC: *r* = 0.85, *p* < 0.01; BMI and BAI: *r* = 0.80, *p* < 0.01; WC and BAI: *r* = 0.61, *p* < 0.01), multivariate analyses were separately adjusted for BMI or WC or BAI or their condition expression (i.e., excess body weight and abdominal obesity). The results are reported as mean with standard deviation (SD), percentages or Beta and 95% confidence interval (CI) (Bootstrap CI, 1000 iterations), unless otherwise indicated. Two-sided *p* values below 0.05 were considered statistically significant.

## Results

The baseline characteristics of the whole sample (*n* = 939) are reported in Table 1. The mean age was 60 years, 78% were in excess body weight, 30% had abdominal obesity, 27% had insulin resistance, 13% were diabetic, 3% had renal failure, and 33% were smokers.

**Table 1:** Characteristics of the study participants

	Total
No. of participants	939
Age (years)	59.7 (6.7)
BMI (kg/m^2^)	27.4 (3.4)
Waist circumference (cm)	98.0 (8.9)
BAI (cm/m^1.5^)	27.8 (3.1)
HOMA index (Unit)	1.9 (2.0)^a^
C-reactive protein (mg/L)	1.7 (2.5)^a^
eGFR (mL/min/1.73 m^2^)	79.4 (1.2)^a^
Leptin (ng/mL)	5.9 (2.1)^a^
White blood cells (10^9^ × L)	6.5 (1.3)^a^
Neutrophils (10^9^ × L)	3.9 (1.4)^a^
Lymphocytes (10^9^ × L)	1.8 (1.4)^a^
Monocytes (10^9^ × L)	0.4 (1.4)^a^
Eosinophils (10^9^ × L)	0.1 (2.1)^a^
Basophils (10^9^ × L)	0.1 (1.6)^a^

Data are expressed as means (SD) or percentage

*BAI* body adiposity index, *BMI* body mass index, *eGFR* estimated glomerular filtration rate, *HOMA* homeostatic model assessment

^a^Geometric mean

**Fig. 1:**
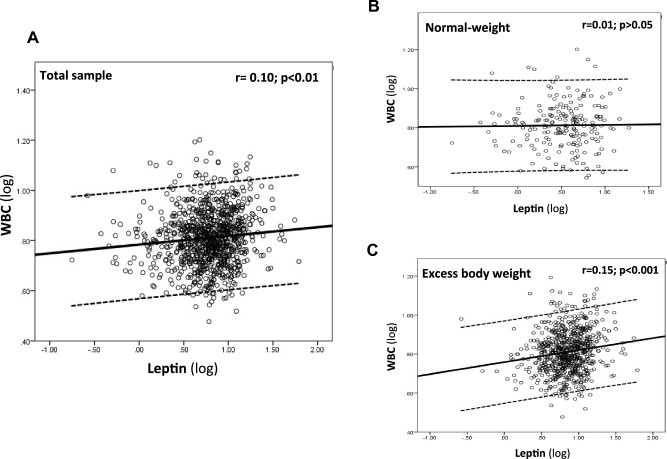
Correlation between Leptin (log-transformed) and white blood cells (WBC, log-transformed) in total sample (**A**), and stratified by normal-weight (**B**) and excess body weight participants (**C**). Solid line is the trend line; dashed lines are the 95% confidence intervals

The analysis of the correlation between WBC and the most relevant characteristics of participants showed a significant and positive association with leptin (*r* = 0.10, *p* < 0.01) (Fig. 1A), CRP (*r* = 0.25, *p* < 0.01), HOMA index (*r* = 0.07, *p* = 0.03), but not with age, renal function and anthropometric indices (*p* > 0.05).

The multivariate analysis confirmed the association between leptin and WBC, after accounting for age, eGFR, HOMA index, CRP, cigarette smoking and hypolipidemic/antidiabetic therapy (Table 2). Separate analyses adjusted for anthropometric indices confirmed the positive and significant trend between leptin and WBC (Table 2), as well as including their conditions in the models (excess body weight—yes or not: *β* 0.10; 95% CI 0.03–0.18; *p* = 0.004; abdominal obesity—yes or not: *β* 0.08; 95% CI 0.01–0.15; *p* = 0.019). Similar results were found by additional analyses also including diabetes (instead of HOMA index and antidiabetic therapy; *β* 0.11; 95% CI 0.05–0.18; *p* = 0.004), or insulin resistance (instead of HOMA index; *β* 0.10; 95% CI 0.03–0.17; *p* = 0.007), or renal damage (instead of eGFR; β 0.11; 95% CI 0.03–0.18; *p* = 0.006).

**Table 2:** Association between leptin levels and white blood cells by linear regression analysis

	Increase in WBC (1-SD log-WBC^e^) β (95% CI^f^)	*p* value
Increase in Leptin (1-SD in log-Leptin^e^) (independent variables)		
Unadjusted	0.09 (0.03–0.16)	0.006
Multivariable model 1^a^	0.08 (0.01–0.16)	0.024
Multivariable model 2^b^	0.09 (0.02–0.17)	0.022
Multivariable model 3^c^	0.09 (0.01–0.18)	0.033
Multivariable model 4^d^	0.09 (0.02–0.16)	0.014

	Increase in lymphocytes (1-SD log-Lymphocytes^e^) β (95% CI^f^)	
Unadjusted	0.12 (0.06–0.18)	0.001
Multivariable model 1^a^	0.12 (0.05–0.18)	0.003
Multivariable model 2^b^	0.09 (0.01–0.17)	0.030
Multivariable model 3^c^	0.11 (0.02–0.19)	0.009
Multivariable model 4^d^	0.09 (0.01–0.16)	0.019

	Increase in monocytes (1-SD log-Monocytes^e^) β (95% CI^f^)	
Unadjusted	0.07 (0.01–0.12)	0.02
Multivariable model 1^a^	0.04 (-0.03–0.12)	0.36
Multivariable model 2^b^	0.03 (-0.06–0.10)	0.52
Multivariable model 3^c^	0.03 (-0.06–0.11)	0.50
Multivariable model 4^d^	0.04 (-0.04–0.12)	0.37

	Increase in eosinophils (1-SD log-Eosinophils^e^) β (95% CI^f^)	
Unadjusted	0.12 (0.05–0.19)	0.001
Multivariable model 1^a^	0.16 (0.08–0.25)	0.001
Multivariable model 2^b^	0.14 (0.05–0.23)	0.007
Multivariable model 3^c^	0.15 (0.05–0.25)	0.006
Multivariable model 4^d^	0.13 (0.03–0.22)	0.006

*BAI* body adiposity index, *BMI* body mass index, *eGFR* estimated glomerular filtration rate, *HOMA* homeostatic model assessment, *WBC* white blood cells

^a^Model 1: adjusted for age, (log)eGFR, (log)HOMA index, (log)CRP, cigarette smoking, hypolipidemic–antidiabetic treatment

^b^Model 2: adjusted for Model 1 plus BMI

^c^Model 3: adjusted for Model 1 plus waist circumference

^d^Model 4: adjusted for Model 1 plus BAI

^e^1-SD log-Leptin = 2.1 ng/mL, 1-SD log-WBC = 1.3*10^9^ × L, 1-SD log-Lymphocytes 1.4*10^9^ × L, 1-SD log-Monocytes 1.4*10^9^ × L, 1-SD log-Eosinophils 2.1*10^9^ × L

^f^Bootstrap confidence intervals (1000 iterations)

Next, we explored the relationship between leptin and WBC subpopulations. There was a positive and significant correlation between leptin and lymphocytes (*r* = 0.12, *p* < 0.001), monocytes (*r* = 0.07, *p* < 0.05) and eosinophils (0.12, *p* < 0.001), but not with neutrophils and basophils (*p* > 0.05).

The linear regression analysis confirmed only the association between leptin and lymphocytes–eosinophils, after adjusting for main potential confounders (Table 2).

In addition, the relationship between WBC and leptin was explored in normal and excess body weight participants, separately. As expected, the individuals with excess body weight had higher anthropometric indices, HOMA index, CRP and leptin, and lower eGFR than normal-weight participants (*p* < 0.05) (Table 3). By contrast, no difference was found in age and WBC—and its subpopulations—(*p* > 0.05).

**Table 3:** Characteristics of the participants stratified by body weight (*n* = 939)

	Normal weight	Excess body weight
No. of participants	202	737
Age (years)	60.0 (6.6)	59.6 (6.6)
BMI (kg/m^2^)	23.0 (1.6)	28.6 (2.7)^b^
Waist circumference (cm)	88.5 (5.9)	100.6 (7.7)^b^
BAI (cm/m^1.5^)	24.5 (2.1)	28.7 (2.7)^b^
HOMA index (U)^‡^	1.2 (1.7)	2.1 (1.9)^b^
C-reactive protein (mg/L)^a^	1.3 (2.6)	1.8 (2.4)^b^
eGFR (mL/min*1.73 m^2^)	83.1 (1.2)	79.4 (1.2)^b^
Leptin (ng/mL)^a^	3.3 (2.2)	7.0 (1.8)^b^
White blood cells (10^9^ × L)^a^	6.5 (1.3)	6.4 (1.3)
Neutrophils (10^9^ × L)^a^	4.0 (1.4)	3.9 (1.4)
Lymphocytes (10^9^ × L)^a^	1.8 (1.4)	1.8 (1.3)
Monocytes (10^9^ × L)^a^	0.3 (1.4)	0.4 (1.5)
Eosinophils (10^9^ × L)^a^	0.1 (2.2)	0.1 (2.1)
Basophils (10^9^ × L)^a^	0.03 (1.6)	0.03 (1.7)

Data are expressed as means (SD)

*BAI* body adiposity index, *BMI* body mass index, *eGFR* estimated glomerular filtration rate, *HOMA* homeostatic model assessment

^a^Geometric mean

^b^Excess body weight vs normal-weight: *p* < 0.01

Given this stratification, a significant association between leptin and WBC was detected only in excess body weight participants (WBC: *r* = 0.15, *p* < 0.001—Fig. 1 B–C; neutrophils: *r* = 0.10, *p* < 0.01; lymphocytes: *r* = 0.13, *p* < 0.001; monocytes: *r* = 0.08, *p* = 0.03; eosinophils: *r* = 0.14, *p* < 0.001). The positive association between leptin and WBC in excess body weight participants was confirmed in an unadjusted model (*β* = 0.16; 95% CI 0.08–0.26, *p* = 0.001) and in a model adjusted for main confounders (*β* = 0.12, 0.03–0.21, *p* = 0.01). The multivariate analysis of WBC subpopulations in excess body weight participants revealed a significant association between leptin and neutrophils (*β* = 0.09, 95% CI: 0.01 to 0.19, *p* < 0.05), lymphocytes (*β* = 0.12, 95% CI 0.03–0.21, *p* = 0.01) and eosinophils (*β* = 0.19, 95% CI 0.08–0.30, *p* = 0.003).

## Discussion

The results of our study indicate a direct relationship between leptin levels and WBC in excess body weight participants, also after accounting for potential confounders, such as age, anthropometric measures, insulin sensitivity, inflammatory markers and renal function. Furthermore, leptin levels were positively associated with some WBC subpopulations, namely monocytes, lymphocytes and eosinophils, but confirmed only with lymphocytes and eosinophils after adjustment for potential confounders, while leptin was associated also with neutrophils in excess body weight participants. Although the subgroup analysis indicated difference in the relationship of leptin–WBC between excess body weight and normal-weight participants, WBC and its subpopulations were not different between the two groups.

To our knowledge, this is the first study directly relating leptin and WBC and its subpopulations, in a relatively large middle-aged sample of general population. The results of this paper are in line with previous studies on the relationship between leptin and WBC [17–21]. However, three of them included a small sample of participants relatively young [19–21], one also obese individuals [21], and another one diabetic patients with nephropathy [18]. In addition, the multivariate models of those studies may be a limitation, indeed covariates such as insulin resistance, renal function, CRP, abdominal circumference or other anthropometric indices alternative to BMI were not considered. In addition, although the difference in leptin between male and female individuals, almost all the studies adjusted only for gender [18–21]. Furthermore, leptin levels were higher than in our sample. This difference may be due to the large homogeneous unselected sample included in our analysis in respect to the selected and small samples of other studies, while leptin of our population was higher than other study of younger and thinner participants [17].

Experimental data support the relationship between leptin and WBC. Indeed, leptin stimulates the proliferation of WBC by direct action on hematopoietic stem cells [19, 30], and it may have a key role on the regulation of the immune system. Leptin may modulate innate immunity by regulation of the activity and function of neutrophils, macrophages, eosinophils, mast cells, and NK cells [9, 10]. On the other hand, leptin may also modulate adaptive immunity by regulation of the activation and proliferation of human T lymphocytes [9, 10, 31]. These effects involve Jak2/STAT3 pathway and SOCS3, and the bidirectional interplay with IL-6, functions that can contribute to the regulation of the production of pro-inflammatory cytokines (e.g., IL-2, TNF-alpha), in particular of those involving in type 2 and eosinophilic inflammation (e.g., IL-4, IL-5).

Given this evidence, leptin may contribute to suboptimal and abnormal immune responses to infections in disorders at higher circulating leptin levels, among which obesity. For example, in influenza infection, leptin resistance is a major infection susceptibility factor in individuals with obesity [32]. Again, in diet-induced-obesity mouse during H1N1 influenza infection, hyperleptinemia was associated with increased mortality, viral spread, and lung inflammation, which were improved by the administration of anti-leptin antibody [33]. In this context, a number of evidence suggested a crucial role of leptin also in SARS-CoV-2 infection [9, 22–24]. Indeed, leptin is associated with cytokine storm during COVID-19 infection in obesity, and it may modulate the gene expression in cardiomyocytes, which may cause myocardial ischemia [34]. Hyperleptinemia may be also associated with thrombotic risk in excess body weight individuals by activation of its receptor on the platelet membrane, that promotes its aggregation [35], thus leading to visceral organ failure [36, 37]. Moreover, leptin was overexpressed in human bronchial epithelial cells during viral infection [38], which in addition to other immune alterations might justify the higher circulating leptin levels in ventilated patients with COVID-19 than control groups [24].

Previous studies showed that leptin was associated with greater monocyte proliferation [39] and activation in COVID-19 cases [40]. Our data confirmed the positive association between leptin and total number of monocytes both in whole population and excess body weight. However, this association was not confirmed after adjustment for the main confounders.

Several experimental and observational studies found a direct relationship between leptin and chronic and acute inflammation [9, 11, 14]. Our data confirmed the positive association between leptin and CRP, as well as reported in previous analysis [41], and the independent association between leptin and WBC was confirmed also after adjustment for that covariate.

Leptin levels could be affected by some classes of drugs. Indeed, it is reduced by the administration of antidiabetic therapy [42] or statins use [43]. Nevertheless, the association between leptin and WBC was confirmed also after adjustment for these classes of drugs. In this context, given the higher leptin levels in some chronic disorders (e.g., rheumatoid arthritis, systemic lupus erythematosus and psoriasis) [11] and in some acute inflammatory conditions during infection, among which the SARS-CoV-2 infection [22–24], the favorable effect of statins use also in both chronic [11, 44] and acute disorders can be hypothesized, as reported in recent studies including COVID-19 patients [45, 46]. However, well-designed randomized controlled trials are needed to confirm this hypothesis and the effect of other drugs affecting circulating leptin levels in different settings.

### Study strengths and limitations

The strengths of our study are: (i) the relatively large general population of men; (ii) the careful standardization of data collection; (iii) the large availability of leptin assessment; (iv) no bias by any pharmacological treatment; (v) the comprehensive covariates included in the models (i.e., age, hypolipidemic–antidiabetic therapy, anthropometric indices, insulin sensitivity, CRP, eGFR, and cigarette smoking).

Nevertheless, the study has also some limitations. The first one is the cross-sectional design; hence, no causal relationship was showed. Albeit the association found between leptin levels and infection susceptibility may be speculative, it is supported by a large cohort, robust results and a large amount of experimental evidence. Another limitation is that our results are generalizable only to a comparable white adult male population. Indeed, there is difference between leptin levels between male and female subjects [47]; likewise also a race difference cannot be ruled out [48]. In addition, a further limitation may be also that the analysis did not directly account for percentage of fat mass. However, other expressions of body fat, strongly associated with circulating leptin levels, were considered in multivariate models (i.e., BAI, BMI, WC or their “condition”—excess body weight and abdominal obesity). Notably, the adjustment for these variables may potentially be also an overadjustment bias, but we aimed to assess the relationship between leptin and WBC independent of the expressions of body fat, and account for other excess body weight effects. Finally, although we account for many covariates, there is still a possibility of residual confounding.

## Conclusions

In conclusion, the results of this analysis indicate that leptin levels are directly associated with WBC in excess body weight adult male participants, independently of anthropometric indices and other potential confounders. This positive association consistent with the previous knowledge concerning this topic supports a crucial and direct role of leptin on modulation of immunity and inflammation in general population, but also in all diseases with concomitant higher leptin levels (e.g., excess body weight, diabetes, hypertension, kidney disease, and autoimmune disorders). This evidence would suggest a role of circulating leptin levels as early marker for susceptibility and severity of chronic and acute inflammation disorders, in addition to that for the cardio-metabolic risk [4–8, 49, 50], hence a potential benefit of leptin-reducing drugs. Further well-designed prospective investigations and intervention studies are needed to confirm these conclusions in different settings.

## Supplementary Information

Below is the link to the electronic supplementary material.

Supplementary file1 (DOCX 29 KB)
